# The Science behind Biomaterials in Female Stress Urinary Incontinence Surgery

**DOI:** 10.1100/tsw.2009.7

**Published:** 2009-01-18

**Authors:** Kaytan V. Amrute, Gopal H. Badlani

**Affiliations:** ^1^ Jacobi Medical Center, Department of Obstetrics and Gynecology, Bronx, NY 10461, United States; ^2^ Wake Forest University Baptist Medical Center, Department of Urology, Department of Urology, Medical Center Boulevard, Winston-Salem, NC 27157, USA

**Keywords:** biomaterials, stress urinary incontinence, autologous grafts, allografts, xenografts, synthetic grafts

## Abstract

Female stress urinary incontinence, while not life threatening, can present with various social and economic implications. Biomaterials, primarily synthetic, are often utilized to augment surgical correction. Repair with biomaterials involves midurethral support to function against weakened connective tissue caused by injury, abnormal collagen metabolism, or genetic predisposition. Even though efficacy rates are high, the potential for complications, such as erosion, are great without comprehension of inherent characteristics of each graft material. Low-weight, macroporous, monofilament synthetic grafts and noncross-linked biologic grafts are examples of biomaterials that implant reasonably well with host tissue. This paper reviews the justification for biomaterial use, host reaction, and the various parameters of natural and synthetic grafts.

## INTRODUCTION

Biomaterials, or graft materials, are used to overcome the drawbacks of conventional surgical procedures for female stress urinary incontinence (SUI) and pelvic organ prolapse (POP). Using native tissue for repair may result in a high rate of recurrence; Kelly plication for stress incontinence has poor long-term outcome compared to other anti-incontinence procedures and recurrence of cystocele is reported in approximately 40% of patients after anterior colporrhaphy[1,2]. Olsen et al. estimated the lifetime risk for a woman to undergo surgery for POP or stress incontinence by age 80 to be 11.2%, with reoperation occurring in close to 30% of cases[3].

SUI is a condition that may significantly affect quality of life and contribute to social debilitation. Either alone (50%) or with urge incontinence (36%), it affects 86% of incontinent women[4]. Treatment costs annually range up to $16 billion (1995 dollars)[5]. In a recent study of 655 women opting for stress incontinence surgery, patients were prepared to pay $118 ± 132/month (approximately $1400–3000/year) for complete resolution of incontinence, with willingness significantly increased in those with higher household income, greater incontinence frequency, and expected incontinence improvement[6]. Optimal outcomes of surgical repair of pelvic floor defects should incorporate several surgical goals, which include restoration of anatomy, preservation of sexual and voiding function, and durability. In order to attain these goals, biomaterials should not replace sound surgical techniques, but should provide reinforcement for long-term efficacy.

"Biomaterial" refers to any biocompatible substance that integrates itself into host tissue and may be naturally derived or synthetic. Natural biomaterials include autologous grafts (native tissue from patient), allografts (i.e., cadaveric), and xenografts (i.e., animal sources). Synthetic biomaterials are generally polymeric grafts that are characterized by absorbability, pore size, and woven structure. Currently, synthetic grafts are used to correct stress incontinence in the majority of cases, but the choice of biomaterial is ultimately dependent on surgeon preference, patient request, and inherent characteristics of the graft material. To limit adverse effects, such as erosion and infection, a thorough knowledge of biomaterial traits is required before use.

## PATHOPHYSIOLOGY OF STRESS INCONTINENCE: BRIEF OVERVIEW

The symptoms and signs of SUI involve a history of the involuntary loss of urine during sneezing, coughing, laughing, or other physical activities that increase intra-abdominal pressure and the observation of such a urinary loss, respectively. Urodynamic stress incontinence is defined as the involuntary loss of urine during an increase in intra-abdominal pressure in the absence of a detrusor contraction[7]. Notable risk factors for SUI include increasing age, parity, history of hysterectomy, and obesity.

Previously, the bladder neck and proximal urethra were believed to be the seat of the continence mechanism. It was hypothesized that with the descent of the proximal urethra (urethral hypermobility), increases in abdominal pressure are transmitted unequally to the bladder and urethra, and when vesical pressure is greater than urethral pressure, stress incontinence occurs. Surgical treatment to address stress incontinence involved retropubic urethropexy or transvaginal suspension of the proximal urethra. Continence is also dependent on the resting urethral resistance or tone. Smooth muscle, skeletal muscle, periurethral vasculature, and intrinsic urethral connective tissue contribute the tone. Intrinsic sphincteric deficiency (ISD) occurs when the urethral sphincter is unable to generate proper resistance for urinary retention[7,8]. Presentation of SUI by patients may either involve ISD or urethral hypermobility or, quite often, both. As such, retropubic urethropexy and transvaginal suspensions may not impart a successful outcome with high continence rates[8].

Subsequent work by Petros and Ulmsten postulated that the midurethra supported by the pubourethral ligament is the primary continence mechanism, and provides the basis for current midurethral sling surgery to repair stress incontinence[9]. The "integral" theory for stress and urge incontinence suggests laxity in the anterior vaginal wall, and its components cause incomplete compression of the urethra and closure of the bladder neck. Furthermore, the laxity may cause activation of the stretch receptors in the bladder neck and proximal urethra, triggering the micturation reflex inappropriately and causing detrusor instability. DeLancey[10] also proposed the "hammock" theory, where the weakness of the underlying and supporting endopelvic fascia of the bladder and urethra causes sphincteric incontinence due to lack of compression of the urethra.

Along with anatomical defects, i.e., weakness of the pubourethral ligament, alterations in collagen and elastin metabolism and genetic predisposition may complicate SUI. Bladder neck and urethral hypermobility is partly determined genetically, as demonstrated in a translabial ultrasound study with nulligravid twin sister pairs with approximately 59% of bladder neck descent variance due to genetic factors[11]. Individuals with connective tissue disorders, such as Ehlers-Danlos and Marfans syndrome, have a higher rate of urinary incontinence. Initial studies conducted in a female population with Ehlers-Danlos showed a 20% higher incidence of SUI compared to a control group[12]. In patients with SUI or severe prolapse, compared to nonaffected individuals, Chen et al.[13] noted increased collagen degradation in vaginal wall tissue with a significant increase in the expression of mRNA of matrix metalloproteinase type 1 (MMP-1), a protease involved in collagen degradation. Concentration of helical peptide α1 (I) 620-633, a urinary collagen degradation product, is significantly higher in patients with SUI compared to those without it[14]. Histopathological differences of skin and endopelvic fascia biopsies in stress incontinent women and unaffected women show reduced collagen content in the incontinent group[15]. Elastin metabolism may also be affected as significantly decreased levels of α1 antitrypsin mRNA, a serine protease inhibitor, were noted in women with stress incontinence and/or prolapse[16]. Elevated elastase activity is evident in the plasma of patients with stress incontinence and provides further evidence of systemic process[17].

## THE "IDEAL" BIOMATERIAL AND HOST REMODELING

Biomaterial use in sling procedures is justified, as it provides midurethral support for continence and contends with anatomical defects, abnormal collagen metabolism, and possible hereditary factors. As such, a paradigm for the "ideal" biomaterial has been expounded[18]. The ideal biomaterial should be one that is sterile, inert, noncarcinogenic, noninflammatory, nonimmunogenic, mechanically durable, nondegradable, inexpensive, readily accessible, and easy to use. Current biomaterials do not meet all of these criteria, but come close to this paradigm[18].

Although biomaterials attempt to be inert and nonimmunogenic, all cause a foreign body response to some extent[19]. Host reaction, initially after implantation, consists of biofilm formation over the graft. Low-molecular-weight proteins, such as albumin, and, later, more complex proteins, such as fibrinogen and extracellular matrix proteins, are incorporated onto the graft. Bacterial contamination may alter the biofilm configuration and may consequently lead to erosion. Immunogenicity of the biofilm occurs through structural changes of the adsorbed proteins, eliciting a typical acute inflammatory response, engaging the complement system, binding of antibodies, and clotting factors and fibrinolysis activation[19].

The chronic inflammatory response then develops: granulation tissue formation with fibroblasts, macrophages, and foreign body giant cells. Neovascularization ensues followed by fibrosis[19]. Macrophages, from extravascular monocytes, are the critical cell type because they play significant roles, such as interaction with lymphocytes and production of mediators that induce protein synthesis and cell proliferation of fibroblasts. Fibroblasts predominate as capillaries and inflammatory cells decrease in number. Consequently, fibrous tissue is formed as collagen and other matrix proteins are deposited[19]. Anomalies in the remodeling process, i.e., bacterial infection or abnormal collagen metabolism, can lead to recurrence, erosion, adhesion formation, and graft shrinkage. Ideally, when a minimal inflammatory response occurs with early infiltration of fibroblasts, rejection of biomaterial is reduced[20].

## NATURAL BIOMATERIALS

Biologic grafts may be harvested from an individual's own fascia (autologous graft), from cadaveric tissue (allograft), or from animal sources (xenograft). Indications for natural biomaterials include allergic reaction to synthetic materials, use in cases involving urethral reconstruction or pelvic bone trauma, history of pelvic irradiation or poor vaginal healing, and surgeon's preference. Advantages include *in vivo* tissue remodeling, histological similarity, and reduced erosion rates. Potential disadvantages are limited supply, cost, unpredictable host tissue integration, inconsistent strength, and possible risk of infection with allografts and xenografts[21].

### Autologous Biomaterials

Autologous grafts are harvested from either of two sites: the rectus abdominis fascia or the tensor fascia lata. While risk of infection is minimal, the risk for potential complications is significant. Harvest from the rectus fascia is constrained secondary to limited length of available fascia, increased morbidity due to incisional pain or wound infection, risk of future abdominal hernia, and increased procedure and recovery times. Previous surgery or irradiation at the harvest site may compromise tissue quality as well. The tensor fascia lata offers greater tensile strength than the rectus fascia, but requires repositioning of the patient, thereby lengthening procedure time. The fascia lata has similar risks for complications, such as acute hematoma, prolonged postoperative pain, and cosmetic issues.

Morgan et al.[22] evaluated 247 women with type II (mainly urethral hypermobility) or type III (i.e., ISD) stress incontinence treated with pubovaginal slings composed of rectus fascia. At a mean follow-up of 51 months, continence rates were 88% overall, 91% for type II and 84% for type III. Secondary procedures, such as transurethral collagen injections or repeat slings, were performed in 14 patients. A low complication rate of 4% was also noted[22]. Another retrospective study showed a 92% overall success rate in 251 patients with a median of 3.1 years follow-up. Permanent urinary retention occurred in four patients, while *de novo* urge incontinence occurred in 3% of patients and persistent urge incontinence in 23%[23]. Similar success rates were demonstrated with autologous fascia lata: Beck et al.[24] revealed a 92% success rate in 170 patients with no erosion complications and minimal *de novo* urgency rates. Another retrospective review using a validated questionnaire showed that 85% of 100 patients reported being dry or improved, and 93% reported being free from pain at the harvest site by the $7^{th}$ day postsurgery. No infections or lower extremity thrombosis were reported[25].

## Allograft Biomaterials

Cadaveric grafts are associated with low risk of disease transmission and erosion. Further advantages include lack of donor site morbidity, shorter operating time, shorter recovery time, and similar efficacy to autologous grafts. Processing techniques and donor factors, however, may contribute to an unpredictable host remodeling and possible resorption[26].

Allograft donors usually consist of an elderly population that may be sedentary, malnourished, and have age-related attenuated fascia[26]. The donors are usually screened first with a thorough medical and social history, followed by serological testing for HIV, Hep B, Hep C, and HTLV-1. After tissue harvest under strict aseptic conditions, removal of antigenic and infectious material occurs via several methods according to the manufacturer. Tissue processing may include freeze drying, after a wash to eradicate various microorganisms. Suspend Tutoplast®, by Colpoplast (Marietta, GA), is an example of a cadaveric graft that undergoes a multistep dehydration method with various organic solvents, followed by sterilization with γ-irradiation.

The quality of allograft biomaterial is determined by processing techniques. Significantly lower tensile strength and stiffness is apparent in freeze-dried fascia compared to autologous grafts and solvent-dehydrated cadaveric fascia[27]. Incomplete rehydration of the allograft of less than 1 h can also affect quality, and irradiation exposure of 20–25 kGy is used to achieve sterility and preserve collagen structure. Irradiation at higher levels damages the tertiary structure of collagen and can affect tensile strength and graft stiffness[26].

Tissue processing techniques and donor factors may explain the range of functional outcomes with allografts in SUI surgery. Success rates of cadaveric fascia used for sling procedures range from 65 to 98%[28]. McBride et al. retrospectively compared autologous slings to Suspend Tutoplast®[29]. Overall, 92.3% of the autologous patients (n = 39) and 90.5% of the allograft patients (n = 32) reported subjective stress continence at 24 months. No differences were noted in bladder neck mobility, maximum urethral closure pressures, and subjective quality-of-life measures. Yet, close to 42% of allograft patients demonstrated urodynamic stress incontinence compared to 0% of autologous patients ($p = 0.007$)[29]. A recent study with a median overall follow-up time of 5.6 years compared autologous rectus fascia (n = 153) to freeze-dried cadaveric fascia (n = 150) in sling procedures[30]. Significantly higher rates of recurrent SUI symptoms and reoperation occurred in the cadaveric group. Adjusting for differences in follow-up time, the cadaveric group revealed high rates of incontinence (16 vs. 5/100 women-years, $p <$ 0.0001) and higher rates of reoperataon (4 vs. 1/100 women-years, $p < 0.0003$)[30].

In brief, data suggest that autologous grafts may be superior to allografts in clinical outcomes of sling procedures. If cadaveric fascia is considered for use, solvent dehydration processing appears to be superior to freeze-dried methods. Unpredictable host integration of allografts is noted to occur and may be related to several factors, such as rate of tissue remodeling, antigenicity of graft material, and high levels of stress placed on the graft during remodeling[31].

## Xenografts

Xenografts are mainly derived from porcine or bovine sources, such as porcine small intestine submucosa, porcine dermis, and bovine pericardium, and act as acellular collagen-based scaffolds. Strict Food and Drug Administration (FDA) guidelines are used to regulate production and take account of information of animal herd, vaccination status, feed source, and bovine spongiform encephalopathy clearance. These implants may be cross-linked; cross-linking between the collagen fibrils serves to protect against resorption by host collagenase degradation[19].

Pelvicol® (Bard Urological Division, Covington, GA) is an example of a porcine dermal implant composed of a flexible sheet of fibrous, acellular collagen and elastin fibers cross-linked by hexamethylene-di-isocyanate (HMDI) followed by $\gamma$-irradiation sterilization. Not only is stability provided against enzymatic degradation, but HMDI cross-linking may cause an attenuated immune response[32]. In the rat model, Pelvicol® induced a slower, but more organized, collagen deposition paralleling the surface of the implant with fewer adhesions compared to synthetic graft material. Encapsulation occurred instead of tissue in-growth, thereby disrupting mechanical retrospective histological study in seven patients with cross-linked porcine dermis undergoing reoperation for sling complications. Explanted specimens demonstrated a trend toward graft preservation with minimal tissue in-growth and remodeling. Cross-linking may prevent cellular infiltration by the host, affecting the remodeling process and leading to graft failure[34]. In those with pubovaginal sling procedures using Pelvicol®, vaginal extrusion was noted in approximately 13 out of 270 patients (4.8%)[35]. A porous version of cross-linked porcine dermis (Pelvisoft®, Bard Urological Division, Covington, GA) is available to promote tissue infiltration and neovascularization. Recently, Guerrero et al. reported a randomized controlled trial comparing autologous fascial sling, Pelvicol®, and TVT; while the sample size in each arm was small, significant differences were noted with lower continence rates and higher reoperation rates in the Pelvicol® group[36].

Noncross-linked porcine dermis (Intexen™ and Intexen LP™, American Medical Systems, Minnetonka, MN) and porcine small intestinal submucosa (SIS) (Surgisis®, Cook Medical, Bloomington, IN) were created to address limitations of cross-linked dermis. The lack of cross-linked collagen fibrils theoretically should facilitate host tissue integration. Histological analysis using Sprague-Dawley male rat models showed a significantly higher degree of cellular infiltration, vascularization, and tensile strength in noncross-linked and porous cross-linked porcine dermis[32].

Acting as a biological scaffold, SIS is obtained from the tunica submucosa of the small intestine and is processed in a manner to remove immunogenic cells and leave intact the complex extracellular matrix (i.e., collagen types II, III, and V) and natural growth factors (i.e., TGF-$\beta$ and FGF-2). It is usually degraded in 4–12 weeks by an active remodeling process that gradually replaces the graft by host connective tissue[19]. Compared to other biomaterials, SIS produces the highest stimulus for collagen fiber formation surrounding the graft[37]. Clinically, in one series of 152 pubovaginal sling patients, a 93% cure rate for SUI was noted at 4-year follow-up, but 50.7% of patients demonstrated urge incontinence[38]. Another retrospective study of 34 patients showed 79% cured of SUI at 2-year follow-up. Minor complications were noted in three patients consisting of suprapubic inflammation, while no cases of erosion or prolonged urinary retention were apparent[39].

## SYNTHETIC BIOMATERIALS

Synthetic grafts, like allografts and xenografts, provide an advantage over autologous grafts by circumventing fascial harvest, thereby reducing operative and recovery times. In addition, synthetic grafts have no risk of infectious disease transmission, have resistance to degradation and long-term preservation of tensile strength, and are readily available and cost effective[40,41]. Synthetics also offer the versatility in size to fit the need for site-specific repair or total repair of all three compartments in addition to the incontinence. Potential disadvantages include failure of remodeling, immunogenicity, and possible host rejection. Examples of polymers used for sling surgery include polyethylene, polytetrafluoroethylene (PTFE), and polypropylene[42].

Synthetic grafts are mainly characterized by pore size, nature of filament fibers, and absorbability. Synthetic meshes are usually created from knitted single-fiber filaments (monofilament) or braided with monofilament yarns into multifilament fibers; tensile strength will vary according fiber type, the weight-to-area ratio, and weave. Fibers may be absorbable or nonabsorbable material, or a combination of both. Pore size is the diameter of open spaces, while interstices are the distance between fibers. Interstices, in multifilament implants, are smaller than pore size and increase contact surface area with the host. These factors ultimately determine whether host inflammatory cells, fibroblasts, and collagen can integrate with the mesh structure. Greater flexibility is apparent in grafts with larger pore size, while grafts that are more interlooped have smaller pores and a higher degree of stiffness[19].

Pore sizes greater than 75 µm are defined as macroporous, while those less than 10 µm are microporous. Macroporous grafts enable admission of the components of the remodeling process: macrophages, fibroblasts, blood vessels, and collagen fibers. Bobyn et al.[42] reported that the best mechanical integration occurred when the pore size was between 50 and 200 µm, with an average of 90 µm. Larger pores (greater than 400–500 µm) limit the remodeling process to the perifilament region, as pores are occupied by adipose cells[43]. Pore size or interstices of less than 10 µm hypothetically allow the passage of bacteria (size of 2 µm or less) and restrict leukocytes (9–15 µm) and macrophages (16–20 µm). As such, clearance of infection and the remodeling process may be limited. A mesh with smaller pores (50 µm or less) or solid product will be encapsulated or induce an amplified foreign body reaction, increasing the risk for erosion[19]. Amid[44] established a classification system for synthetic grafts used in abdominal herniorrhaphy based on porosity and fiber type. Meshes composed of macroporous/ monofilament fibers are categorized as Type I (i.e., polypropylene), microporous/multifilament fibers as Type II (i.e., expanded PTFE), and macroporous and microporous/multifilament fibers as Type III (i.e., polyethylene tetraphthalate).

Based on the principles of host tissue remodeling and infection prevention, Type I macroporous monofilament synthetic mesh is currently used in most stress incontinence procedures, most often polypropylene. Early experiences with Types II and III meshes showed significant complications[41]. Rates of *de novo* urgency after Mersilene sling implantation approached 20% in several reports, while rates of erosion and excision were approximately 17%[45,46]. Gore-tex, or expanded PTFE, becomes encapsulated *in vivo* and excision rates range from 3 to 30%, as the full-length slings have higher rates compared to patch slings[47,48]. Marlex, a single weave of monofilament polypropylene that is macroporous, is a heavier (95 g/m$^2$) and less flexible example of polypropylene mesh; erosion has been noted in 7% of patients[49].

Polypropylene, the current polymer used in most midurethral sling products today, is composed of two monofilaments of polypropylene and contains a larger pore size (1500 µm) to deliver a greater degree of flexibility. Reported extrusion rates for polypropylene mesh products range from 0.4 to 4.8% for TVT (Gynecare, Johnson & Johnson, NJ), 1–10.5% for SPARC (American Medical Systems, Minnetonka, MN), and 0–6.7% for MONARC (American Medical Systems, Minnetonka, MN)[50,51,52]. ObTape™ (Mentor-Porges, Le Plessis Robinson, France) and UraTape® are two microporous, multifilament polypropylene meshes that are nonwoven, nonknitted, and have been thermally bonded; the two differ in that the latter has a suburethral silicone-coated section. Used in sling procedures via the transobturator route, the two have similarly high rates of erosion and are no longer utilized[40,53].

While studies regarding natural biomaterials and sexual function are lacking, recent research has evaluated sexual function and synthetic mesh placement. However, these studies are limited by small sample size and, in some cases, use of nonvalidated questionnaires. In their retrospective study, Elzevier and colleagues[54] demonstrated no significant overall adverse effect in sexual function after TVT placement; however, the study did not use a validated questionnaire and was subject to recall bias. Using a validated questionnaire in patients with distal suburethral polypropylene mesh placement with a mean follow-up time of 19.4 months, Shah et al. examined 25 out of 29 sexually active patients that demonstrated no significant difference in desire, arousal, lubrication, orgasm, satisfaction, and pain pre- and postoperatively[55]. In another study evaluating the use of transvaginal nonabsorbable mesh in pelvic organ prolapse surgery, the authors showed that there was no significant impairment or improvement in sexual function 1 year postoperatively[56].

## CONCLUSIONS

Biomaterials have a definite role in the repair of SUI. As utilization increases, escalation of adverse events can be tempered with familiarity of the host remodeling process and the characteristics of various graft materials. Successful outcomes are dependent on proper graft integration into host tissue. Neovascularization, collagen in-growth, and fibrous tissue formation should occur without infection or significant inflammatory reaction. Poorly integrated grafts include cross-linked xenografts and synthetic microporous grafts that may become encapsulated, leading to hardening or shrinkage. Examples of well-integrated biomaterials are noncross-linked biologic grafts and, in terms of synthetics, large-pore, low-weight, monofilament mesh[57]. In conclusion, to limit potential complications, use of biomaterials should be judicious and individualized according to patient need and surgeon's experience.
